# Tamoxifen Delivery System Based on PEGylated Magnetic MCM-41 Silica

**DOI:** 10.3390/molecules25215129

**Published:** 2020-11-04

**Authors:** Margarita Popova, Neli Koseva, Ivalina Trendafilova, Hristina Lazarova, Violeta Mitova, Judith Mihály, Denitsa Momekova, Georgi Momekov, Iskra Z. Koleva, Hristiyan A. Aleksandrov, Georgi N. Vayssilov, Ágnes Szegedi

**Affiliations:** 1 Institute of Organic Chemistry with Centre of Phytochemistry, Bulgarian Academy of Sciences, 1113 Sofia, Bulgaria; ivtrendafilova@gmail.com (I.T.); lazarova@orgchm.bas.bg (H.L.); 2 Institute of Polymers, Bulgarian Academy of Sciences, 1113 Sofia, Bulgaria; mitova@polymer.bas.bg; 3 Research Centre for Natural Sciences, Institute of Materials and Environmental Chemistry, 1117 Budapest, Magyar Tudósok Körútja 2, Hungary; judith.mihaly@ttk.hu (J.M.); szegedi.agnes@ttk.mta.hu (Á.S.); 4 Faculty of Pharmacy, Medical University of Sofia, 1000 Sofia, Bulgaria; dmomekova@yahoo.com (D.M.); gmomekov@gmail.com (G.M.); 5 Faculty of Chemistry and Pharmacy, University of Sofia, 1126 Sofia, Bulgaria; ohik@chem.uni-sofia.bg (I.Z.K.); haa@chem.uni-sofia.bg (H.A.A.); gnv@chem.uni-sofia.bg (G.N.V.)

**Keywords:** tamoxifen, mesoporous magnetic nanoparticles, PEGylation, release properties, DFT calculations

## Abstract

Magnetic iron oxide containing MCM-41 silica (MM) with ~300 nm particle size was developed. The MM material before or after template removal was modified with NH_2_- or COOH-groups and then grafted with PEG chains. The anticancer drug tamoxifen was loaded into the organic groups’ modified and PEGylated nanoparticles by an incipient wetness impregnation procedure. The amount of loaded drug and the release properties depend on whether modification of the nanoparticles was performed before or after the template removal step. The parent and drug-loaded samples were characterized by XRD, N_2_ physisorption, thermal gravimetric analysis, and ATR FT-IR spectroscopy. ATR FT-IR spectroscopic data and density functional theory (DFT) calculations supported the interaction between the mesoporous silica surface and tamoxifen molecules and pointed out that the drug molecule interacts more strongly with the silicate surface terminated by silanol groups than with the surface modified with carboxyl groups. A sustained tamoxifen release profile was obtained by an in vitro experiment at pH = 7.0 for the PEGylated formulation modified by COOH groups after the template removal. Free drug and formulated tamoxifen samples were further investigated for antiproliferative activity against MCF-7 cells.

## 1. Introduction

Mesoporous silicas are promising drug carriers because of their beneficial textural characteristics, such as uniform pores with controllable size, large pore volume, high specific surface area (>700 m^2^/g), and good chemical and thermal stability [1,2,3]. During the past decade, mesoporous silica nanoparticles have been used for the delivery of a wide variety of chemotherapeutic and bioimaging agents owing to their unique characteristics and tailored methods of preparation. A drawback of the conventional drug delivery carriers used in oncology is their inability to control the release rate and simultaneously provide site-specific delivery. The application of superparamagnetic iron oxide nanoparticles is an advanced approach to developing targeted antitumor drug delivery systems. The modification of magnetic nanoparticles or their polymer-embedded varieties permits selective accumulation of the drug to the targeted organ or tissue of the body by applying a sufficiently strong magnetic field [4,5,6]. Usually the efficient magnetite/mesoporous silica composites possess a high surface area (500–700 m^2^/g) [7,8,9,10,11], which is important for the highest amount of loaded drug and for the controlled release properties at the target place. Magnetic carriers offer some advantages such as mechanical and chemical stability in the bioenvironment of the obtained formulations. The high magnetic moment of the functionalized carriers is among the most important requirements for successful applications in biomedicine, especially for magnetic targeting of anticancer drugs.

**Scheme 1:**
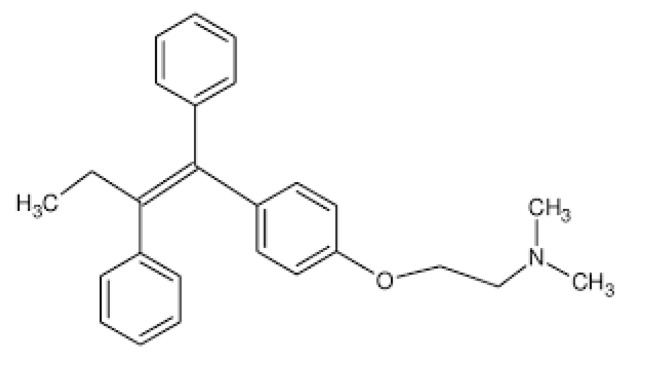
Tamoxifen molecule.

Tamoxifen [*Z*-1-(4-β-dimethylaminoethoxy-phenyl)-1,2-diphenylbut-1-ene] (Scheme 1) (TX), a nonsteroidal selective estrogen receptor modulator (SERM), is a first-line drug in the treatment of breast cancer [12,13,14,15,16]. Tamoxifen has antagonist effects against estrogen receptors and thus reduces DNA synthesis and responsiveness of cancer cells to estrogen stimulatory effects, thereby promoting cell death. In addition, tamoxifen prevents tumor growth by stimulating the tumor-inhibiting transforming growth factor B (TGFb) and downregulating the tumor-stimulating insulin-like growth factor 1 (IGF-1) [14]. Tamoxifen (TX) is prescribed during different stages of breast cancer, including the prevention stage for women at high-risk or the early stages of cancer [15]. Despite its beneficial effects against breast cancer, long-term treatment with tamoxifen is obstructed by the tamoxifen-related induction of endometrial and liver cancers [14]. In order to minimize these side effects, tamoxifen formulation into nanoparticulated carriers can be used to deliver the required dose to the tumor site and thus to reduce the exposure on healthy tissues [15,16]. In addition, nanosized carriers can protect hydrophobic tamoxifen from macrophages scavenging during transportation within the blood and thus prolong its systemic circulation and sufficient accumulation in tumor tissue [17,18].

In this study, new composite carriers based on magnetic mesoporous silica with 300 nm nanoparticles, modified with -NH_2_ or -COOH groups and then PEGylated were developed and loaded with tamoxifen as targeted delivery systems. The influence of the sequence of template removal and mesoporous silica modification on the drug-loading efficiency and release profile was also studied. In order to clarify the interaction of the drug molecules with the carrier density, functional calculations of relevant model systems were performed.

## 2. Results and Discussion

### 2.1. Textural and Spectroscopic Characterization

**Figure 1:**
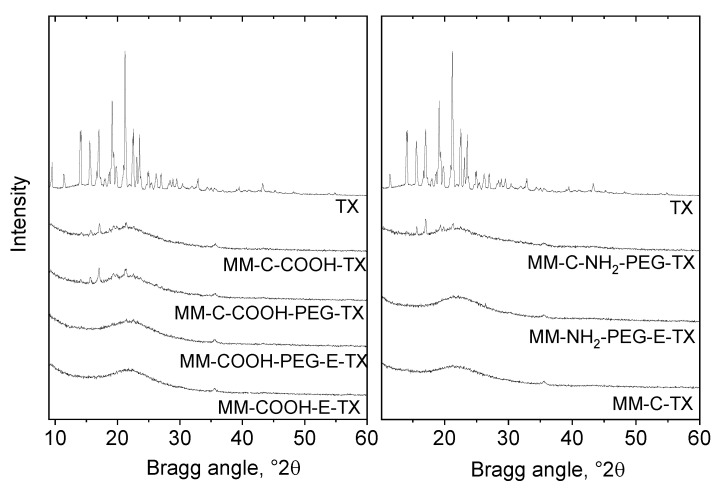
XRD patterns of the MCM-41 silica nanoparticles with magnetic iron oxides (MM) sample and its amino/PEG- or carboxylic/PEG-modified varieties loaded with tamoxifen.

MCM-41 silica nanoparticles with magnetic iron oxides (MM) were characterized by X-ray powder diffraction and the results can be found in Figure 1. An XRD pattern of dried iron oxide nanoparticles shows the presence of magnetic iron oxides (Figure S1). Kim et al. [19] developed an X-ray diffraction method for the differentiation of commercial maghemite and magnetite minerals based on the detailed profile analysis of [511] reflection at around 57 2θ°. The method is based on the small shift of 511 reflection only in one phase, i.e., magnetite (Fe_3_O_4_) of maghemite toward higher 2θ° values compared to magnetite when the full widths at half maximum (FWHM) of the two compounds make it possible to differentiate their overlapping. The XRD patterns of the nanosized magnetic iron oxide particles are shown in Figure S1. Iron oxide nanoparticles show widened reflections typical of small nanoparticles. The pattern can be identified rather as a maghemite structure than as magnetite. The particle size calculated by the Sherrer equation applying the profile fitting method is 8 nm. The formation of superparamagnetic nanoparticles with ferromagnetic behavior was proven by magnetization measurements as well. The saturation magnetization of initial iron oxide nanoparticles is 59 emu/g and corresponds to the presence of maghemite. The saturation magnetization value of MM material (6 emu/g) corresponds to the low amount of iron in the sample (4%) (Figure S2). Magnetization data thus supported that silica particles with sufficient magnetic field response were produced, making the developed mesoporous silica composite suitable for successful application as a drug carrier. The successful magnetic particles incorporation was evidenced by TEM images (Figure S3).

Reflections are widened due to the high dispersion of the iron oxide. The crystallite size calculated by the Sherrer equation applying the profile fitting method is about 20 nm. Reflections typical of tamoxifen (TX) with low intensity were detected on the TX-loaded formulations modified with COOH/NH_2_-groups and PEGylated after the template removal (MM-C-COOH-TX, MM-C-NH_2_-TX, MM-C-COOH-PEG-TX, MM-C-NH_2_-PEG-TX), indicating that a small part of crystalline tamoxifen is deposited on the external surface of the carrier. The lack of tamoxifen reflections in the formulations modified with COOH/NH_2_-groups and PEGylated before the template removal is a proof for its amorphous state on the carrier (Figure 1). It seems that the applied tamoxifen-loading method plus the removal of the template before the modification of the carrier resulted in the deposition of the drug mainly in the pores of the silica, preventing its crystallization.

**Figure 2:**
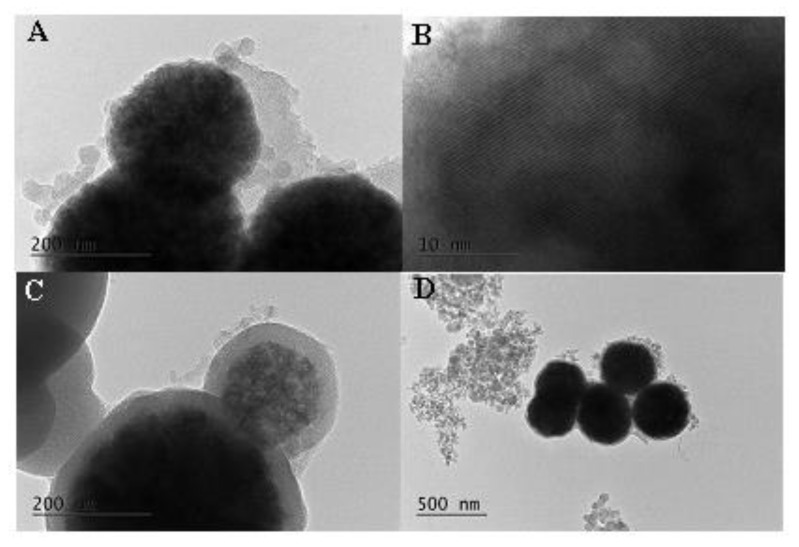
TEM images of the studied materials: (**A**,**B**)—MM; (**C**,**D**)—MM-PEG.

Formation of spherical MM nanoparticles with size around 300 nm was registered by TEM (Figure 2A,B).

Existence of ordered mesopores was also supported by TEM images. The presence of iron oxide nanoparticles can also be detected. The polymer layer around the silica particles for PEG-modified formulations is clearly seen in Figure 2C,D. The particle size of the PEG-modified MM is around 400 nm in comparison to the 300 nm initial MM nanoparticles.

**Figure 3:**
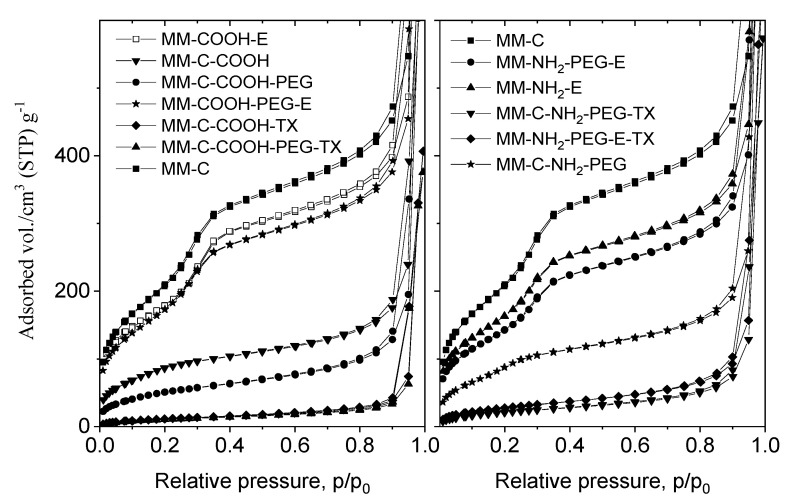
N_2_ physisorption isotherms of the MM sample and tamoxifen-loaded formulations.

The mesoporous structure of the MM composite was proven by N_2_ physisorption measurements (Figure 3).

The nitrogen physisorption experiments of the parent and modified samples were performed after the template removal by calcination or extraction. The textural parameters of all samples are summarized in Table 1. The obtained composites show type IV isotherms without hysteresis loop, typical for the MCM-41 mesoporous structure. Modification with COOH/NH_2_-groups after the template removal results in significant decrease of surface area (826 m^2^/g for MM to 313 m^2^/g for MM-C-COOH and 560 m^2^/g for MM-C-NH_2_). When the template was removed after organic modification, the specific surface area decrease was lower (688 m^2^/g for MM-COOH-E and 647 m^2^/g for MM-NH_2_-E) (Table 1).

The PEGylation procedure also leads to some surface area decrease, which is more pronounced for the COOH/NH_2_-modified samples prepared after template removal. The tamoxifen loading leads to a further decrease in surface area and pore volume due to the penetration of drug into the mesopores. The nitrogen physisorption data of the COOH-modified samples (MM-C-COOH-TX and MM-C-COOH-PEG-TX) (Figure 3) show very low surface area and pore volume, indicating pore blocking. The surface areas of the MM-COOH-PEG-E-TX and MM-NH_2_-PEG-E-TX samples are higher than those of their counterparts, the MM-C-COOH-PEG-TX and MM-C-NH_2_-PEG-TX samples. The reason can be low or no drug deposition in the pores of the carrier for the former samples.

**Scheme 2:**
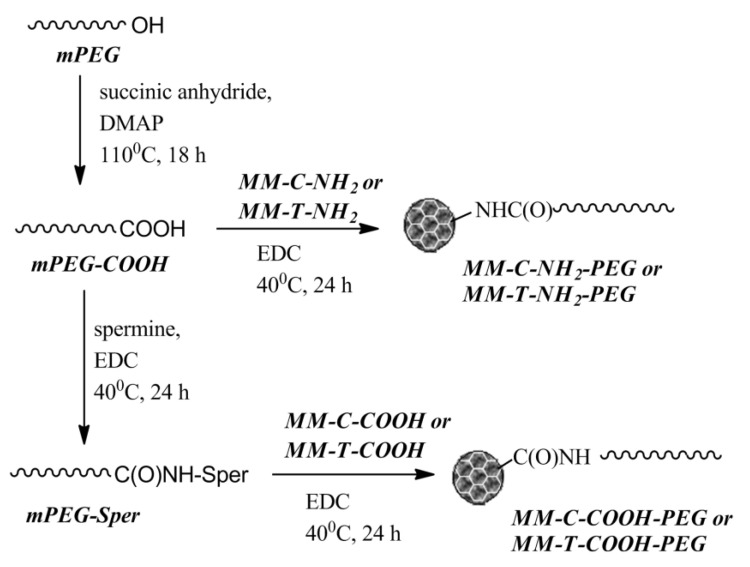
Synthetic steps to obtaining PEGylated mesoporous nanoparticles. DMAP: 4-(dimethylamino)pyridine; EDC: N-(3-dimethylaminopropyl)-N′-ethylcarbodiimide hydrochloride.

The biocompatibility of modified MM nanoparticles was increased via surface grafting of PEG chains. Two PEGylating agents were synthesized from poly(ethylene glycol) monomethyl ether (mPEG) with molar mass 5000 g/mol. mPEG functionalized with a carboxylic acid end group (mPEG-COOH) was prepared via the reaction of mPEG with succinic anhydride in the presence of 4-(dimethylamino)pyridine (DMAP). Whereas, aminofunctionalized mPEG was obtained using the natural polyamine spermine and N-(3-dimethylaminopropyl)-N′-ethylcarbodiimide hydrochloride (EDC) via a standard procedure for formation of an amide bond with mPEG-COOH. The product was denoted as mPEG-Sper, indicating that the polymer chain was bearing spermine residue (Scheme 2).

The same procedure was applied for the PEGylation of mesoporous nanoparticles. The MM-C-NH_2_ and MM-T-NH_2_ materials were reacted with mPEG-COOH to yield PEGylated nanoparticles via formation of an amide linkage between the surface amino groups and the polymer chain. Similarly, the MM-C-COOH and MM-T-COOH nanoparticles were grafted with an mPEG-Sper via a spermine bridge (Scheme 2).

The TG analyses were used for the determination of the amounts of the formed organic groups. In MM-C-NH_2_ (11.2 wt. %) and MM-C-COOH (16.4 wt. %) they are higher than those formed onto the MM-NH_2_-E (6.0 wt. %) and MM-COOH-E (9.6 wt. %) samples extracted after modification (Table 1). This effect is due to the modification with NH_2_/COOH groups only on the external surface of MM-T. Further, PEGylation of MM-T-NH_2_ and MM-T-COOH resulted in the formation of a thinner PEG layer than that formed around the MM-C-NH_2_ and MM-C-COOH samples calcined before modification. The template removal procedure applied after the modifications decreases the amount of modifiers (NH_2_/COOH and PEG) because of their partial leaching during the template extraction. Despite the sequence of modification procedure and template removal, high drug loading was detected. The highest amount of tamoxifen could be loaded on the MM-COOH-E-TX (31.4 wt. %) and MM-NH_2_-E-TX (26.6 wt. %) samples in comparison to the MM-C-COOH-TX (24.2 wt. %) and MM-C-NH_2_-TX samples (19.4 wt. %). The higher surface area of the MM-COOH-PEG-E and MM-NH_2_-PEG-E samples than those of the MM-C-COOH-PEG and MM-C-NH_2_-PEG samples leads to the predominant drug deposition in the pores, which could be concluded also by the significant decrease in the surface area and pore volume after tamoxifen loading, detected by N_2_ physisorption data (Figure 3, Table 1).

### 2.2. Computational Modeling of the Interaction of Tamoxifen with the Nanocarrier

#### 2.2.1. Modeling of the Isolated Tamoxifen Molecule

**Figure 4:**
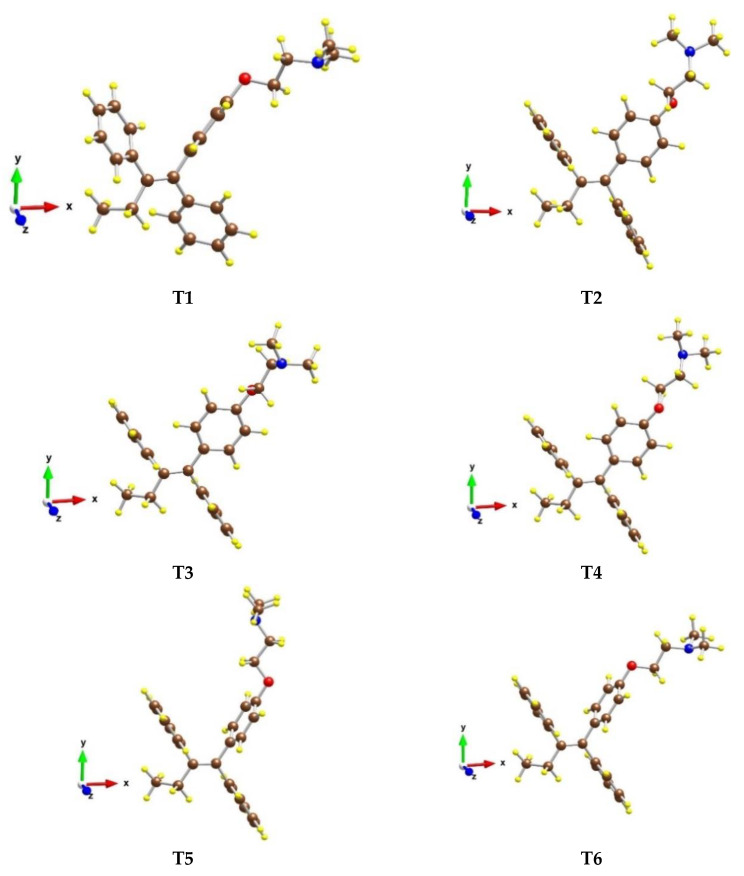
Optimized structures of different conformers of the tamoxifen molecule in gas phase. Color coding: C—brown, O—red, N—blue, and H—yellow.

Six conformers of the tamoxifen molecule were optimized, in which the relative positions of the three benzene rings (Figure 4) as well as of the ethyl, ethoxy, and -N(CH_3_)_2_ groups were different. The most stable structure is T1 (see Table S1), while the other models are less stable by 13–29 kJ/mol.

In model T1, the two phenyl groups, which are in trans position, are almost perpendicular to each other, while in the other conformers both groups are parallel. Structure T2 is next in stability, as it is less stable by 13 kJ/mol than T1. Structures T3 and T4 have similar stability as both are by 24 kJ/mol less favorable than structure T1. Their geometries differ only by the position of the O(CH_2_)_2_N(CH_3_)_2_ part.

#### 2.2.2. Adsorption on Silanol (SiOH)-Modified Silicate Surface

**Figure 5:**
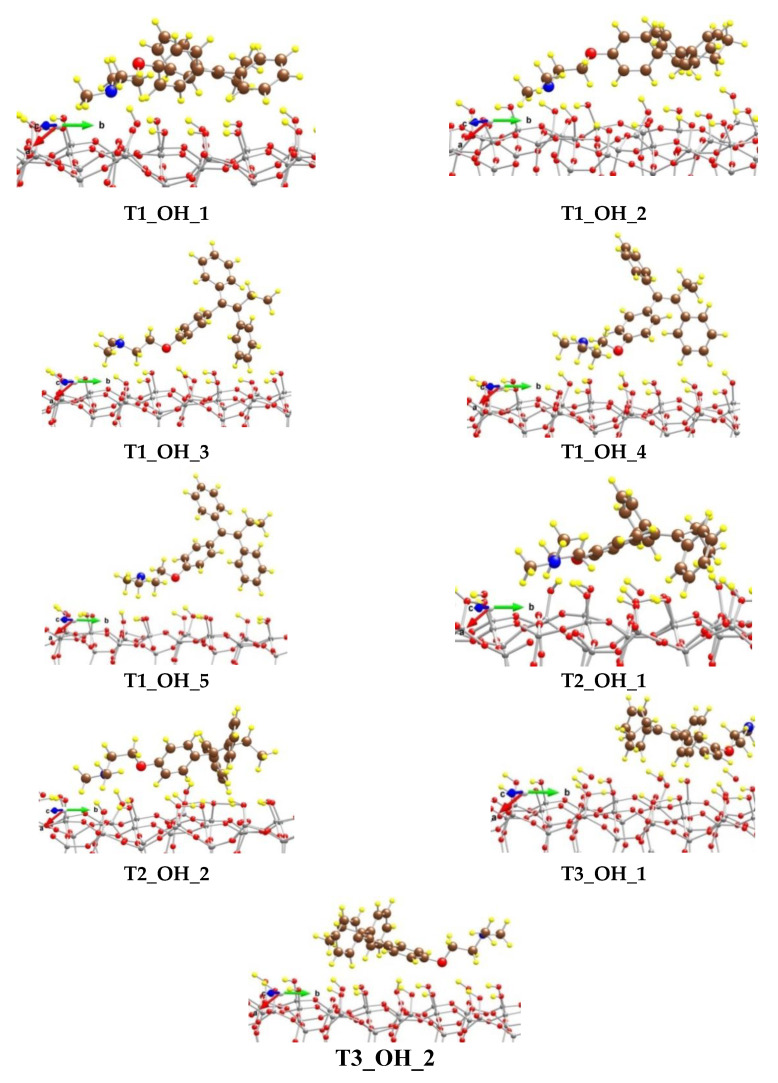
Optimized complexes of a tamoxifen molecule adsorbed on (SiOH)-terminated silicate surface. Color coding: Si—gray, C—brown, O—red, N—blue, and H—yellow. For visual clarity the O center from tamoxifen is represented with a larger sphere.

We modeled adsorption complexes not only with the T1 conformer of tamoxifen, but also with the T2 and T3 structures (Figure 5). Interestingly, the most stable model, structure T2_OH_1, is not obtained by adsorption of the most stable conformer T1, but by the T2 conformer (Table S1).

All typical interactions between tamoxifen and the substrate are observed in the T2_OH_1 complex. In it, two strong hydrogen bonds were observed: (1) between a phenoxy O atom and a H atom from a silanol group with a length of 189 pm and (2) between N from the amino group and a H atom from a silanol group, as the corresponding N-H distance is 161 pm. In total, six H atoms from the three benzene rings interact with the surface O centers from the SiOH groups as the corresponding O-H distances are in the range 232–263 pm, as some of phenyl H centers interact with two O centers. The H atom from the methylene group next to the phenoxy fragment interacts with O from the SiOH group as the distance is 260 pm. In addition, H atoms from the N(CH_3_)_2_ methyl groups as well as from the ethyl part next to the phenyl groups interact with the silanol groups as the corresponding O-H distances are in the region 237–263 pm. The binding energy of tamoxifen is −279 kJ/mol, which is 95 kJ/mol higher than the calculated binding energy (BE) of the drug molecule on the surface covered by the carboxyl groups (see below). Structure T2_OH_2 is only 14 kJ/mol less stable than the T2_OH_1 model, resulting in lowering of the BE of the drug molecule by the same value. The N atom from the N(CH_3_)_2_ group is bound to a silanol H atom as the hydrogen bond length is 156 pm. The O atom from the tamoxifen molecule weakly interacts with two surface protons at distances of 269 and 291 pm. H atoms from the phenyl groups are coordinated to the silanol groups as the distances of the sixth closest contacts vary from 215 to 293 pm.

Among the structures with the adsorbed T1 conformer, the most stable model is T1_OH_1, which is less favorable by 52 kJ/mol than T2_OH_1 with a BE of T1 conformer of −214 kJ/mol. The other four models considered from the series are 84–192 kJ/mol less stable than T2_OH_1, as the BE values of T1 are in the range −182 to −75 kJ/mol. In the different structures the individual hydrogen bonds and other interactions vary significantly, as can be seen in Table S1.

The adsorption of the T3 conformer is not so favorable, as the obtained complexes, T3_OH_1 and T3_OH_2, are less stable by 131 and 151 kJ/mol, respectively, than the T2_OH_1 model.

#### 2.2.3. Adsorption on CH_2_COOH-Modified Silicate Surface

The theoretical experiments were performed only for COOH-modified silica because we hypothesized that in this case a stronger interaction between the drug and silica surface will occur compared to the nonmodified silica. Modification with NH_2_-groups could provide additional experimental data and insight about the state of the drug loaded in the porous material as a basis for comparison with the COOH-modified material. We modeled a CH_2_COOH-modified silicate surface, as SiOH were replaced by SiCH_2_COOH. In the surface model, formation of hydrogen bonds between the oxygen atoms of the carbonyl group and the protons of an adjacent carboxyl group is observed.

**Figure 6:**
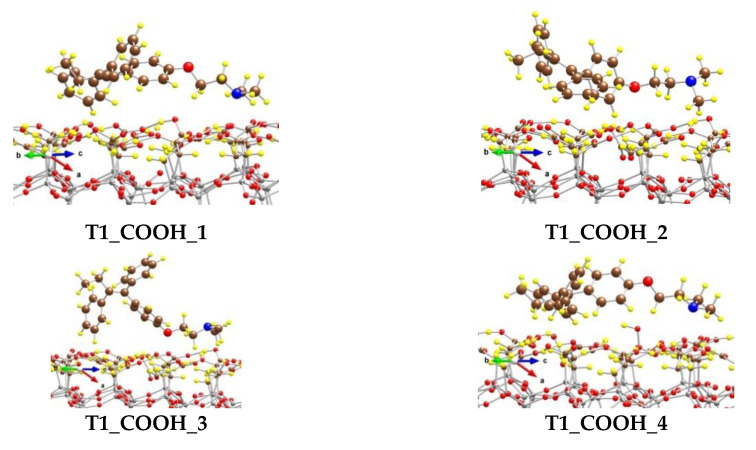

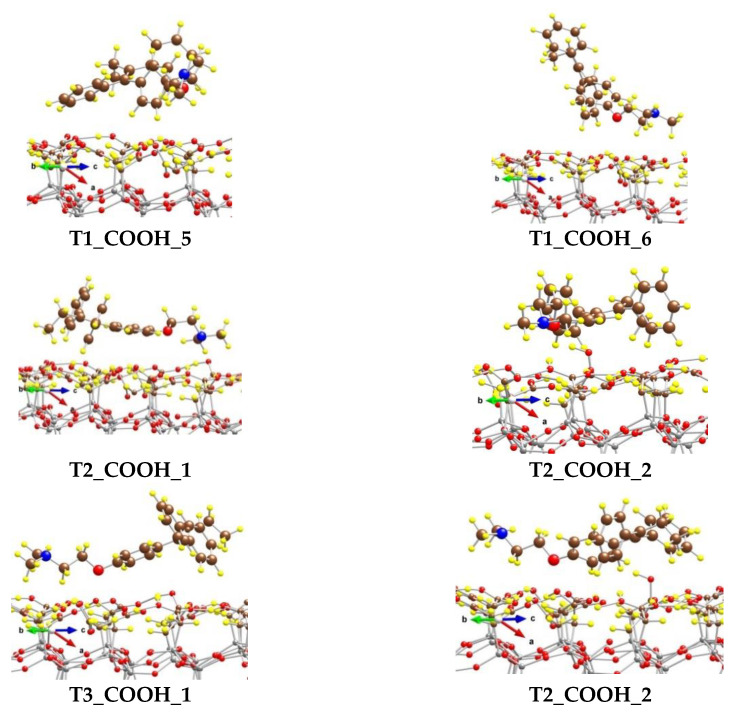
Optimized complexes of a tamoxifen molecule adsorbed on a (CH_2_COOH)-terminated silicate surface. Color coding: Si—gray, C—brown, O—red, N—blue, and H—yellow. For visual clarity the C and O centers from tamoxifen are represented with larger spheres.

We modeled several adsorption complexes of the most stable conformer of tamoxifen T1, which differs by the orientation and the position of the tamoxifen molecule on the CH_2_COOH-modified silicate surface (Figure 6).

In the most stable one, T1_COOH_1, some of the H atoms from the benzene rings (five in total) interact with the O centers from the OH moieties of the surface carboxyl groups, as the distances for the six shortest contacts vary from 238 to 293 pm. Four H centers from the N(CH_3_)_2_ group also form weak bonds with O atoms from the -CH_2_COOH moieties, as the shortest distance is 243 pm. Both H atoms from the methylene group next to the O atom in tamoxifen interact weakly with the surface O at distances of 275 and 292 pm. Although the O atom from the tamoxifen molecule could form a hydrogen bond with an atom H from the carboxyl groups, it is too far from the surface and does not interact with them. The binding energy (BE) of the adsorbed tamoxifen molecule in this complex is −184 kJ/mol.

The other complexes, T1_COOH_2 to T1_COOH_6, are 40 to 124 kJ/mol less stable than the T1_COOH_1 model, due to different types of interactions between the drug and the support. Respectively, the BE values for those complexes are lower by the same amount.

We also modeled complexes with the next two in stability conformers—T2 and T3. Model T2_COOH_1 is 36 kJ/mol less favorable than T1_COOH_1 and the binding energy with respect to the structure T2 is −161 kJ/mol. The tamoxifen molecule is coordinated to the surface via four H atoms from the phenyl groups as the distances vary from 235 to 301 pm. H atoms from the N(CH_3_)_2_ group also interact with O centers from the COOH groups and the O-H distances are in the range 255–298 pm. In the other model, T2_COOH_2, the O atom from the adsorbate is bound to the surface H atom and the hydrogen bond length is 186 pm. Six weak interactions between H from benzene rings and O from carboxyl groups are also formed with distances of 246–286 pm. Additionally, the H atoms from the methyl groups bound to nitrogen interact with the surface as the H–O(COOH) distances are 247, 264, and 269 pm. This model is 54 kJ/mol less stable than T2_COOH_1. Models T3_COOH_1 and T3_COOH_2 are 69 and 78 kJ/mol less stable than T1_COOH_1, as the binding energies of the T3 conformer of tamoxifen are −138 and −130 kJ/mol, respectively.

In summary, our results showed that tamoxifen molecule interacts more strongly with the silicate surface terminated by silanol groups compared to the one modified with CH_2_COOH groups, as the BE values for the most stable complexes in both cases are −279 kJ/mol and −184 kJ/mol, respectively. In the case of the surface modified with carboxylic groups, the most favorable adsorption complex is formed with the T1 conformer unlike the other surface, where the adsorption of the T2 conformer is more favorable. The crucial factor for the stability of the adsorption complexes of tamoxifen with the silicate surface terminated by silanol groups seems to be the existence of an interaction between the N center from the amino group and a H atom from a silanol group, while the stability of the complexes with surface modified by –COOH groups depends on the number and strength of the hydrogen bonds formed between H atoms from the drug molecules and O centers from the carboxyl group.

### 2.3. Experimental and Calculated Vibrational Frequencies

**Figure 7:**
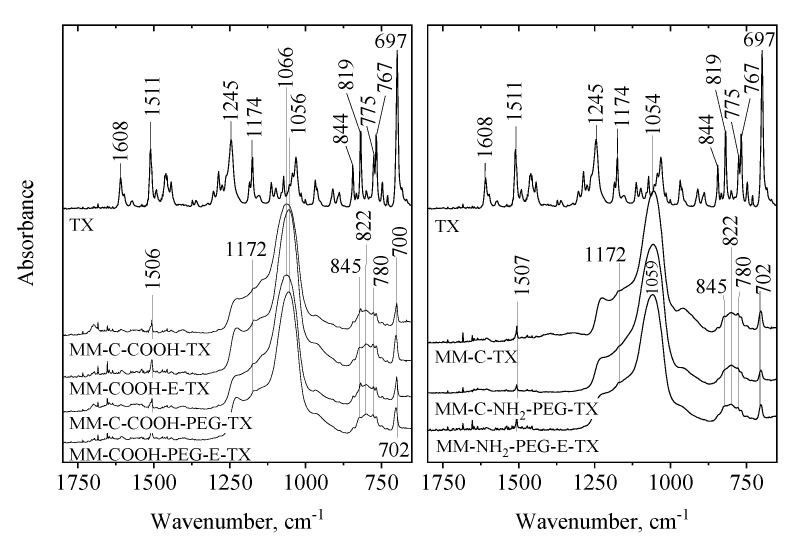
ATR FT-IR spectra of pure tamoxifen and tamoxifen-loaded MM and NH_2_-, COOH and PEGylated samples.

The interactions between magnetic silica carriers and tamoxifen molecules were studied by ATR FT-IR spectroscopy (Figure 7). The fingerprint region of the tamoxifen IR spectrum shows characteristic bands of aliphatic C=C (at 1608 cm^−1^) and of ring C=C (at 1511 cm^−1^) stretching vibrations [20]. Bands at 1245 and 1174 cm^−1^ can be assigned to C-O/C-N stretchings, while bands from the spectral region of 650–900 cm^−1^ typically belong to C-H bendings [21].

The calculated vibrational frequencies of the isolated and adsorbed tamoxifen molecule, presented in Table S2, in general support the experimental observations. For the T1 conformer, the stretching vibrations of C-H bonds in phenyl groups are in the region of 3091–3143 cm^−1^, while the C–H antisymmetric and symmetric stretching vibrations of the methylene and methyl groups are in the range of 2816–3048 cm^−1^ (the region is not shown in the experimental spectrum in Figure 7). The calculated stretching vibration of the alkene C=C bond located between the benzene rings is at 1609 cm^−1^ compared to 1608 cm^−1^ in the experimental spectrum. The stretching vibrations in the aromatic rings are calculated in the ranges 1474–1593 cm^−1^ and 1324–1428 cm^−1^. The experimental band at 1511 cm^−1^ assigned to ring C=C falls into the former range. The bending vibrations of CH_2_ (scissoring) and CH_3_ groups (asymmetric and symmetric) are calculated at 1351–1464 cm^−1^. The calculated out-of-plane C-H bending vibrations of benzene rings are located in the region 686–981 cm^−1^ in agreement with the corresponding experimentally observed bands at 650–900 cm^−1^. The C-O stretchings, C(Ph)-O and C(CH_2_)-O at 1220 and 1008 cm^−1^, respectively, appear at the same region as the C-N stretching vibrations, which are at 1260, 1174, 1041, and 1034 cm^−1^. In the experimental spectrum the C-O and C-N stretchings are observed at 1245 and 1174 cm^−1^. The results for the other two conformers which we considered, T2 and T3, are very similar to these for T1, discussed above.

The spectra of the drug-loaded MM silica matrices are dominated by the strong Si-O-Si vibration around 1045 cm^−1^. However, weak bands related to tamoxifen can be witnessed, too. It is worth noting that the ring C=C stretching band is shifted to a lower frequency whereas the ArC-H deformations are shifted to a higher frequency, indicating that a weak interaction between the drug and the silica matrix might exist. No significant difference was observed with PEGylation. The amino-modification before template removal treatment induces some small change in the structure of the silica matrix, indicated by the peak shift of the Si-O-Si matrix (from 1045 to 1059 cm^−1^).

When MM-C-COOH was used as a drug carrier, the same phenomena could be observed. Again, the shift of the ring C=C stretching band (from 1511 to 1506 cm^−1^) and that of the ring C-H bendings suggest the interaction between tamoxifen molecules and the silica matrix. The COOH-modification before template removal caused structural change in the silica matrix (shift of Si-O-Si band from 1066 to 1056 cm^−1^). We have to note, however, that for the COOH-modified samples, the strongest tamoxifen band at 697 cm^−1^, assigned to ArCH ring deformation, is less shifted (697 to 700 cm^−1^) compared to the shift observed for the MM-C-TX (697 to 702 cm^−1^). It seems that the interactions of the aromatic protons of the drug molecule with the unmodified silica matrix are more favorable than the ones with the COOH-modified surfaces. These experimental trends have been supported by the density functional theory (DFT) calculations of modeling the interactions between the tamoxifen conformers and carrier surface, as described above. When PEG is also present, probably the CH and C-O-C moieties enhance the drug-carrier interaction, presumably by H-bonding-type interaction.

In general, adsorption of the tamoxifen molecule on both modified silicate surfaces does not significantly change the vibrational frequencies of the drug molecule. The experimentally found changes in the IR bands are within the accuracy of the calculated frequencies. In all cases, the vibrational frequency of C(Ph)-O stretching, 1210–1240 cm^−1^, appears at higher frequencies than this for C(CH_2_)-O stretching, 1000–1040 cm^−1^. The C-N stretching frequencies are at the same spectral range at around 1260, 1180, and 1040 cm^−1^. The C=C stretching vibration is at ~1600 cm^−1^, while the carbon-carbon stretching vibrations of the aromatic rings are at 1470–1595 cm^−1^. They partly overlap with the bending vibrations of methylene and methyl groups, 1330–1430 cm^−1^. The out-of-plane CH bending vibrations of the benzene rings are at the range of 670–990 cm^−1^.

### 2.4. In Vitro Release of Tamoxifen

**Figure 8:**
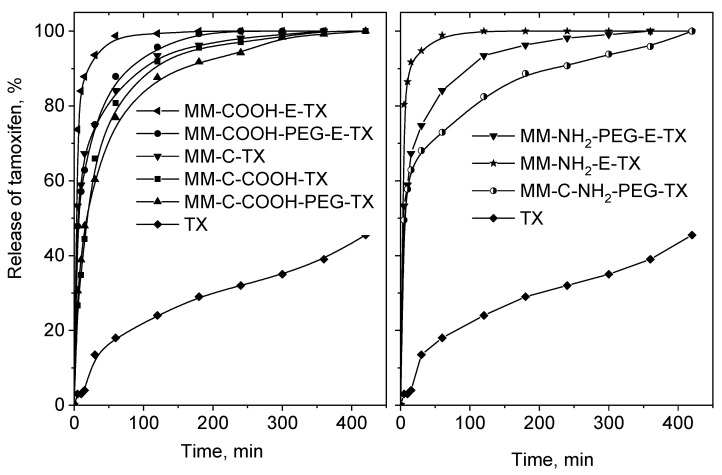
In vitro release of pure tamoxifen at pH = 7.0, and that of the tamoxifen-loaded MM composites.

In vitro release process of pure tamoxifen and the mesoporous MM-silica-loaded varieties was studied at pH = 7 (Figure 8).

Free tamoxifen was poorly dissolved in the studied 8 h (45 wt. %). All formulations show better tamoxifen release because of its amorphization and incorporation into the channel system of the silica matrix. The MM-COOH-E-TX and MM-NH_2_-E-TX samples show burst release of tamoxifen, which is in good agreement with the spectroscopic and theoretical data indicating weaker drug–support interaction. The stronger interaction between tamoxifen and silanol groups of MM resulted in slower drug release. Total release of the loaded tamoxifen was achieved in 7 h for all formulations. Modification by COOH/NH_2_ groups and grafting of PEG chains resulted in a significant decrease of the tamoxifen release rate. This effect is more pronounced when the modifications and PEGylation were performed after the template removal due to the formation of a thicker PEG layer (4.0 wt. % and 4.6 wt. % for MM-COOH-PEG-E and MM-NH_2_-PEG-E, respectively compared to 9.4 wt. % and 8.9 wt. % for MM-C-COOH-PEG and MM-C-NH_2_-PEG, respectively). The optimal modification extent (content of NH_2_/COOH groups and PEG layer, Table 1) in the case of the MM-C-COOH-PEG-TX and MM-C-NH_2_-PEG-TX samples is responsible for the sustained tamoxifen release. The release of iron oxide nanoparticles was not observed during the drug release experiments, supporting their successful incorporation in the silica matrix.

### 2.5. Cytotoxicity Study

**Figure 9:**
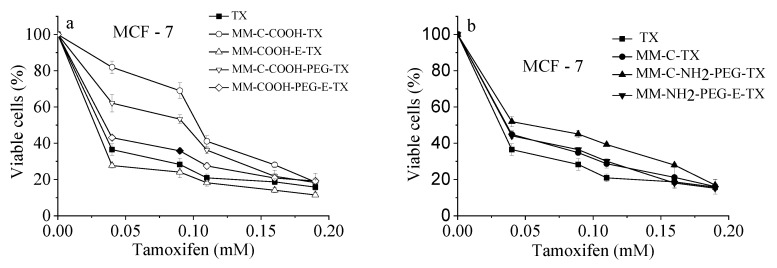
Cytotoxicity of free tamoxifen drug and its silica-loaded varieties: (**a**) COOH-modified and (**b**) NH_2_-modified nanoporous MM silica composites after 72 h continuous exposure at 37 °C. Each data point represents the arithmetic mean ± SD of 6 separate experiments.

A comparative investigation of the cytotoxic effect of tamoxifen loaded into NH_2_- and COOH-modified and/or PEGylated mesoporous silicas vs. free drug (as ethanol solution) was performed [22,23]. For the sake of fullness, the anticancer cytotoxicity bioassay was also performed for alternative PEG-coated counterparts. The growth inhibitory concentration–response curves are shown on Figure 9 and the corresponding equieffective IC_50_ values are summarized in Table 2.

Results obtained showed that both free drug and its modified mesoporous silica-based formulations evoked strong, concentration-dependent inhibition of the growth of cultured tumor cells comparable with free tamoxifen. Juxtaposition of the concentration–response curves clearly indicates that the encapsulation of the anticancer drug did not compromise its antineoplastic activity. The exception of this tendency showed COOH-modified samples, modified after template removal, namely: MM-C-COOH-TX and MM-C-COOH-PEG-TX. In these cases, the concentration–response curves were shifted towards higher doses (Figure 9). These observations were corroborated by the calculated IC_50_ values (Table 2), which were almost two- and three-times higher as compared to free-drug or other loaded formulations, respectively. These facts correlate well with the release profiles whereas slower tamoxifen release was encountered from formulations prepared with template removal before modification with COOH/NH_2_ groups and PEGylation procedure.

In the interest of clarity, the cytotoxic potential of non-loaded silica carriers was also evaluated. The MCF-7 cells as well as normal mouse fibroblast (CCL-1) were treated with the same concentration of carriers as those in the drug-loaded samples. The corresponding dose–response curves are shown in Figure S4. As evidenced, the silica nanocomposites are devoid of cytotoxic activity against both tested cell lines since no suppression of the vitality of the treated cells (values in the 76–98% range) was observed. Thus, the formerly observed antiproliferative effects of the loaded formulations are due to the presence of tamoxifen only.

## 3. Materials and Methods

### 3.1. Materials

FeCl_2_∙4H_2_O, FeCl_3_∙6H_2_O, hexadecyl trimethyl ammonium bromide (CTAB), tetraethyl orthosilicate (TEOS), and tamoxifen ≥99% (TX) were provided by Sigma-Aldrich (Darmstadt, Germany). Poly(ethylene glycol) monomethyl ether (mPEG, Mw = 5000), 4-(dimethylamino)pyridine (DMAP), succinic anhydride (SA), N-(3-dimethylaminopropyl)-N′-ethylcarbodiimide hydrochloride (EDC), spermine, dichloromethane (DCM), toluene, and chloroform were purchased from Sigma-Aldrich.

### 3.2. Synthesis of Magnetic Iron Oxide Nanoparticles

Magnetic iron oxide nanoparticles were synthesized by a coprecipitation procedure of ferric and ferrous ions containing salts [24]. The two iron salts, 0.01 mol FeCl_2_∙4H_2_O and 0.02 mol FeCl_3_∙6H_2_O, were mixed at room temperature in 100 mL degassed, distilled water and stirred until the total dissolution of the salts. Two grams of NaOH (0.8 mol) dissolved in 100 mL degassed, distilled water were added to the iron salts solution until a final pH = 12 was recorded. The solution was further stirred at 500 rpm for 3 h under nitrogen gas flow. The obtained solid product was centrifuged and washed with distilled water until it was chloride free. The nanoparticles were dried at room temperature.

### 3.3. Synthesis of the Magnetic Porous Silica Nanocarriers and Their Modification with NH_2_ and COOH Groups

Spherical MCM-41 silica with 300-nm-sized magnetic particles were prepared according to the procedure of Huh et al. [25] with some modifications. This sol-gel procedure was carried out at 80 °C without cosolvent, only in water solution, and applying NaOH as a catalyst. The relative molar composition of the reaction mixture was 1 TEOS:0.12 C_16_TMABr:0.31 NaOH:1190 H_2_O. The formed gel was aged at 80 °C for 2 h, then washed with distilled water until neutral pH, and dried at ambient temperature. Template removal from a fraction of the synthesized MCM-41 material was carried out at 500 °C with a rate of 1 °C/min for 5 h. The solid product was decanted and washed with ethanol and water, then dried at room temperature. The synthesized material containing the template was denoted as MM-T, and that with removed template via calcination, MM-C.

#### Modification with NH_2_ and COOH Groups

In a typical procedure, 1 g of MM-T or MM-C, formerly dried at 150 °C for 2 h, was mixed with 100 mL abs. ethanol and reacted with 2 mL of 3-aminopropyltriethoxysilane (APTES) for 5 h at 60 °C by stirring. The functionalized sample was separated by filtration and washed 3 times with 10 mL of ethanol, then dried at room temperature. The samples were dried by vacuum evaporation (0.04 Pa) at room temperature for 6 h and denoted as MM-NH_2_ and MM-C-NH_2_.

Modification with COOH-groups was performed on the already prepared nanoparticles MM-C-NH_2_ and MM-NH_2_. The incorporation of the COOH group was done through the reaction with succinic anhydride in toluene. To remove adsorbed water, azeotropic drying of 1 g amino-modified silicas was carried out at 115 °C with 20 mL of anhydrous toluene. Succinic anhydride (6.6 mmol) was added to the mixture at 60 °C and treated for 24 h. The samples were dried by vacuum evaporation (0.04 Pa) at room temperature for 6 h and denoted as MM-COOH and MM-C-COOH.

The organic template was removed from the MM-NH_2_ and MM-COOH samples by extraction procedure with ethanol at 80 °C. The solid product was decanted and washed with ethanol and water, then dried at room temperature. Thus, prepared materials were denoted as MM-NH_2_-E and MM-COOH-E.

### 3.4. Preparation of the PEGylated Magnetic Silica Nanoparticles

#### 3.4.1. Synthesis of Methoxypoly(ethylene glycol)-carboxyl Functionalized (mPEG-COOH)

mPEG (5.11 g, 0.001 mol) was dissolved in toluene (30 mL) and dried by distilling off at least of 2/3 of the solvent. Succinic anhydride (0.1534 g, 0.0015 mol) and DMAP (0.102 g, 0.001 mol) were added and the mixture was stirred for 18 h at 110 °C. The reaction mixture was dissolved in CH_2_Cl_2_, filtered to remove the unreacted SA, and the polymer was precipitated in diethyl ether. The product was dissolved in an adequate amount of acetone and dialyzed (MWCO: 1000) against a mixture of acetone/deionized water (1:1) for 24 h then against deionized water for 2 days. The dialyzed solution was lyophilized to obtain mPEG-COOH with 91% yield.

NMR (600 MHz, CDCl_3_) δ: 4.40 (4H, t, -CH_2_CH_2_OC(O)-), 3.59 (450H, m, (-CH_2_CH_2_O-), 3.31 (3H, s, CH_3_O-), 2.60–2.55 (4H, m, -CH_2_CH_2_C(O)OH).

#### 3.4.2. Synthesis of Methoxypoly(ethylene glycol)-amino Functionalized (mPEG-Sper)

mPEG-COOH (1.0042 g, 0.0002 mol), spermine (0.041 g, 0.0002 mol), and EDC (0.038 g, 0.0002 mol) were dissolved in 10 mL CHCl_3_ and the mixture was stirred for 24 h at 40 °C. The solvent was removed by evaporation and the product was dissolved in an adequate amount of deionized water and dialyzed (MWCO: 1000) for 3 days. The dialyzed solution was lyophilized to obtain mPEG-Sper with 85% yield.

NMR (600 MHz, CDCl_3_) δ: 4.40 (4H, t, -CH_2_CH_2_OC(O)-), 3.59 (450H, m, (-CH_2_CH_2_O-), 3.31 (3H, s, CH_3_O-), 3.3–3.2 (4H, m, -CH_2_N(CO)CH_2_-), 3.1–3.0 (4H, m, NH_2_CH_2_-), 2.9–2.8 (4H, m, -CH_2_NHCH_2_-), 2.7–2.6 (4H, m, -NCH_2_CH_2_CH_2_N-), 2.60–2.55 (4H, m, -CH_2_CH_2_C(O)OH), 2.2–2.0 (4H, m, -NCH_2_CH_2_CH_2_CH_2_N-).

#### 3.4.3. PEGylation of Aminomodified Nanoparticles

MM-C-NH_2_ nanoparticles (0.300 g), mPEG-COOH (0.300 g), and EDC (0.0115 g) were mixed in 5 mL CHCl_3_ and stirred for 24 h at 40 °C. The particles were washed three times with CHCl_3_ to remove unreacted PEG-COOH. The product was isolated by centrifugation at 12,000 rpm and dried under reduced pressure. The same procedure was used for PEGylation of MM-T-NH_2_ nanoparticles. The synthesized materials were denoted as MM-C-NH_2_-PEG (yield 0.315 g) and MM-T-NH_2_-PEG (yield 0.317 g). The amount of grafted PEG was determined by thermogravimetric analysis.

#### 3.4.4. PEGylation of COOH-Modified Nanoparticles

MM-C-COOH nanoparticles (0.200 g), mPEG-Sper (0.200 g), and EDC (0.007 g) were mixed in 5 mL CHCl_3_ and stirred for 24 h at 40 °C. The mixture was washed three times with CHCl_3_ to remove unreacted mPEG-Sper. The product was isolated by centrifugation at 12,000 rpm and dried under reduced pressure. The same procedure was used for PEGylation of MM-T-COOH nanoparticles. The synthesized materials were denoted as MM-C-COOH-PEG (yield 0.208 g) and MM-T-COOH-PEG (yield 0.206 g). The amount of grafted PEG was determined by thermogravimetric analysis.

The organic template was removed from the MM-T-NH_2_-PEG and MM-T-COOH-PEG samples by extraction procedure with ethanol at 80 °C. The solid product was decanted and washed with ethanol and water, then dried at room temperature. Thus, prepared materials were denoted as MM-NH_2_-PEG-E and MM-COOH-PEG-E.

### 3.5. Tamoxifen Loading

Portions of 0.100 g of initial and modified MM materials and tamoxifen in a weight ratio of 1:0.5 were stirred in 1 mL ethanol until the total evaporation of the solvent. Then the powdered products were washed 3 times with 5 mL water. Samples were dried at 40 °C overnight. The tamoxifen-loaded MM materials were designated as MM-C-TX, MM-C-COOH-TX, MM-C-NH_2_-TX, MM-COOH-E-TX, MM-NH_2_-E-TX, MM-C-COOH-PEG-TX, MM-C-NH_2_-PEG-TX, MM-COOH-E-PEG-TX, and MM-NH_2_-E-PEG-TX materials. The sequence of fragments in the abbreviations corresponds to the experimental steps to the nanoparticles’ preparation, where C stands for template removal by calcination and E for template removal by extraction.

### 3.6. Characterization

X-ray powder diffraction patterns were recorded by a Bruker D8 Advance diffractometer (Bruker, Germany) with Cu Kα radiation and a LynxEye detector with constant step of 0.02° 2θ and counting time of 17.5 s per step. Mean crystallite sizes were determined by the Topas-4.2 software.

Nitrogen physisorption measurements were carried out at −196 °C using AUTOSORB iQ-C-MP-AG-AG (Quantachrome Instruments (Anton Paar brand), Boynton Beach, FL 33426, USA). Before adsorption analysis, silica samples were outgassed under high vacuum (<10^−6^ mbar) at 250 °C for 2 h, whereas functional-group-modified, PEGylated, and drug-loaded ones were pretreated at 80 °C for 5 h. The specific surface area was calculated by the BET equation between the 0.01–0.2 relative pressures. Pore size distribution was evaluated by the BJH method.

TEM images were taken on a JEOL JEM 2100 TEM (200 kV) (JEOL, Tokyo 196-8558, Japan). The samples were suspended in a small amount of ethanol and a drop of the suspension was deposited onto a copper grid covered by carbon supporting film and dried at ambient temperature.

Thermogravimetric measurements were performed by an STA449F5 Jupiter instrument (NETZSCH Gerätebau GmbH, Selb, Germany) with a heating rate of 5 °C/min in air flow.

FT-IR spectra were recorded by means of an IRAffinity-1 “Shimadzu” Fourier Transform Infrared (FTIR) spectrophotometer with MIRacle Attenuated Total Reflectance device (diamond crystal; depth of penetration of the IR beam into the sample is approximately 2 µm; (PIKE Technologies, Madison WI 53719, USA)) in the range 600–4000 cm^−1^ with a resolution of 4 cm^−1^. The spectra were corrected for H_2_O and CO_2_ using the IR solution software program.

The magnetic properties of the iron oxide nanoparticles were measured using a Quantum Design MPMS-5S superconducting quantum interference device (SQUID) magnetometer (San Diego, CA 92121, USA). The dried samples were packed into gelatin capsules in vacuum grease. Zero-field-cooled–field-cooled (ZFC–FC) magnetization curves were obtained from −268 to 25 °C. In ZFC mode the sample was cooled in a nominally zero magnetic field (in practice in about -1 Oe) from 25 to −268 °C and the magnetization was subsequently measured in a field of 10 Oe with increasing temperature. In FC mode the sample was cooled in 10 Oe from 25 to −268 °C and the magnetization was subsequently measured in the same field with increasing temperature. Hysteresis loops of dried samples were taken in fields between ± 50 kOe at −268 °C and 25 °C. The saturation magnetization (Ms) was defined as the value of the magnetization at 2 T.

^1^H-NMR spectra were measured on a Bruker Avance II+ 600 NMR spectrometer (Bruker, Germany) at a temperature of 20 °C in CDCl_3_.

### 3.7. Computational Details

Our calculations were performed with VASP program [26,27] based on the density functional theory (DFT) under periodic boundary conditions. We used the exchange-correlation function proposed by Perdew–Burke–Ernzerhof (PBE) [28] with additional Grimme dispersion correction [29] and PAW pseudopotentials [30,31]. The cutoff energy of plane-wave basis is 400 eV. The Brillouin zone was sampled using the Γ point only. The geometry relaxation was performed until all forces became smaller than 5 × 10^−4^ eV/pm. The same computational method was used for calculation of the vibrational frequencies of the drug molecules. They were obtained from a normal mode analysis where the elements of the Hessian were approximated as finite differences of analytical gradients, displacing each atomic center by 3.0 pm either way along each direction.

The employed slab model of a silicate surface consists of two layers of an infinitely extended β-cristobalite(111) surface with parameters of the unit cell as follows: a = 43 Å; b = c = 20.36 Å; α = β = 90°; γ = 135°. Each layer of the slab model contains 32 (8 × 4) Si atoms. A vacuum gap of more than 1 nm was added to avoid an interaction between the neighboring surface images.

The terminal groups on both sides of the slab models are silanol (SiOH), but we also built a model in which the silanol groups are replaced with SiCH_2_COOH from one of the sides of the slab, the one which interacts with the tamoxifen molecule.

The binding energies (BE) of the adsorbed tamoxifen molecule on the silicate surface were calculated as BE = E_T_/_S_ − E_S_ − E_T_, where E_T_ is the total energy of the corresponding conformer of isolated tamoxifen molecule denoted in the name of the structure, E_S_ is the total energy of the silicate surface, and E_T/S_ is the total energy of the surface model together with the adsorbed tamoxifen molecule. Negative values of BE indicate that the interaction between the drug molecule and the substrate is energetically favorable (exothermic).

### 3.8. In Vitro Release of Tamoxifen

For in vitro release studies, 10 mg of the drug-loaded silica nanoparticles were incubated in 50 mL phosphate buffered saline (PBS pH 7.0) at 37 °C under stirring (100 rpm). At appropriate time intervals, 2 mL samples were withdrawn and replaced by fresh buffer. The withdrawn samples were centrifuged at 15,000 rpm for 15 min and the concentration of tamoxifen released into the supernatant was determined by UV-Vis spectroscopy at a wavelength of 276 nm.

### 3.9. Cell Lines and Culture Conditions

The cytotoxicity assessment of free and tamoxifen-loaded mesoporous silica carriers was carried out against MCF-7 human cell line (estrogen-receptor-positive, breast-cancer-derived cells) (German Collection of Microorganisms and Cell Cultures (DSMZ GmbH, Braunschweig, Germany)) and CCL-1, American-type cell culture (ATCC). The cells were cultured in a controlled environment—cell culture flasks at 37 °C in an incubator ‘BB 16-Function Line’ Heraeus (Kendro, Hanau, Germany) with humidified atmosphere and 5% CO_2_. The growth medium was 90% RPMI-1640 + 10% FBS + 1 mmol sodium pyruvate and 10 µm/mL human insulin.

### 3.10. Cytotoxicity Assessment (MTT-Dye Reduction Assay)

The cellular viability after exposure to free tamoxifen or its mesoporous silica formulations was assessed using the standard MTT-dye reduction assay as described elsewhere [22] with slight modifications [23]. The method is founded on the reduction of the yellow tetrazolium salt MTT to a violet MTT-formazan via the mitochondrial succinate dehydrogenase in viable cells. In short, exponentially growing cells were seeded in 96-well, flat-bottomed microplates (100 μL/well) at a density of 1 × 10^5^ cells per mL. After 24 h incubation at 37 °C, they were exposed to various concentrations of the tested formulations or free drug for 72 h. For each concentration a set of 6 wells was used. Following the treatment time, 10 μL MTT solution (10 mg/mL in PBS) aliquots were added to each well. Afterwards, the microplates were incubated for a further 4 h at 37 °C and the formed MTT-formazan crystals were dissolved by addition of 100 μL of 2-propanol acidified with 5% formic acid. The MTT-formazan absorption was recorded using a LabeximLMR-1 microplate reader at 580 nm. Cell survival fractions were calculated as a percentage of the untreated control. In addition, IC_50_ values were derived from the concentration–response curves.

## 4. Conclusions

Spherical composite mesoporous MCM-41-type silica with magnetic particle sizes around 300 nm was synthesized. The pore surface of the material was functionalized by incorporation of NH_2_- and -COOH groups and further PEGylated. XRD investigations showed that during the synthesis of the composite, the magnetic nanoparticles were efficiently incorporated into the silica spheres. The developed nanoparticulate materials were studied as tamoxifen carriers. Loading of tamoxifen in amino/carboxylic-modified and PEGylated MCM-41 nanoparticles resulted in about 19.4–31.4 wt. % of drug content, which is deposited inside the pores of the silica carrier. Textural characterizations also proved that the loaded anticancer tamoxifen drug was localized in the channels of the mesoporous matrix. Spectroscopic and theoretical data suggest weaker bonding between the COOH groups of the mesoporous silica nanoparticles and tamoxifen molecules, and stronger interaction between silanol groups and drug molecules. In vitro release of tamoxifen at pH = 7 was modified, reaching total drug release within 7 h for all systems. The sustained tamoxifen release was ensured by optimal modification extent (content of NH_2_/COOH groups and PEG layer) of the mesoporous composite carrier after its calcination.

## Figures and Tables

**Table 1:** Textural characteristics of the initial MM, MS-NH_2_, and the tamoxifen-loaded MS-NH_2_ samples.

Samples	Surface Area, m^2^/g	Pore Volume, cm^3^/g	Pore Diameter, nm	Content of NH_2_/COOH or PEG, wt. %	Drug Content, wt. %
MM-C	826	2.30	2.5	-	-
MM-C-TX	141	1.61	1.0	-	22.0
MM-C-NH_2_	560	1.58	2.1	11.2 *	-
MM-C-NH_2_-PEG	337	1.36	2.0	11.2 * (8.9) **	-
MM-C-NH_2_-PEG-TX	78	0.89	1.0	11.2 * (8.9) **	19.4
MM-NH_2_-E	647	1.89	2.5	6.0 *	-
MM-NH_2_-PEG-E	562	1.74	2.5	6.0 * (4.6) **	-
MM-NH_2_-PEG-E-TX	104	1.03	1.0	6.0 * (4.6) **	26.6
MM-C-COOH	313	1.27	1.0	16.4 *	-
MM-C-COOH-TX	44	0.63	1.0	16.4 *	27.0
MM-C-COOH-PEG-E	183	1.13	1.0	16.4 * (9.4) **	-
MM-C-COOH-PEG-TX	40	0.58	1.0	16.4 * (9.4) **	24.2
MM-COOH-E	688	1.98	2.5	9.6 *	-
MM-COOH-E-TX	90	1.01	1.0	9.6 *	31.4
MM-COOH-PEG-E	645	1.82	2.5	9.6 * (4.0) **	-
MM-COOH-PEG-E-TX	102	1.05	1.0	9.6 * (4.0) **	28.0

* content of NH_2_/COOH groups; ** content of PEG.

**Table 2:** Equieffective (IC_50_) values.

	Cell Line	MCF-7
Sample		IC_50_(mM)
TX		0.029
MM-C-TX		0.035
MM-C-NH_2_-PEG-TX		0.035
MM-NH_2_-E-PEG-TX		0.051
MM-C-COOH-TX		0.038
MM-COOH-E-TX		0.010
MM-C-COOH-PEG-TX		0.026
MM-COOH-PEG-E-TX		0.035

## Supplementary Materials

The following are available online. Figure S1: XRD of the iron oxide nanoparticles; Figure S2. Magnetic properties of iron oxide nanoparticles and MM composite; Figure S3: TEM images of MM composite; Figure S4: Cytotoxicity of the studied silica carriers against MCF-7 and CCL-1 cells after 72 h continuous exposure at 37 °C. Each data point represents the arithmetic mean ± SD of 6 separate experiments; Table S1: Relative and binding energies (in kJ/mol) and selected distances (in pm) for the modeled structures of tamoxifen complexes with silicate surfaces terminated by silanol groups or modified with -CH_2_COOH groups; Table S2: Selected calculated vibrational frequencies for three of the modeled structures of tamoxifen conformers and their complexes with silicate surfaces terminated by silanol groups or modified with -CH_2_COOH groups. The most stable complexes are marked with bold.

## References

[1] Vallet-Regi M., Rámila A., Del Real R.P., Pérez-Pariente J. (2001). A new property of MCM-41: Drug delivery system. Chem. Mater..

[2] Wang S. (2009). Ordered mesoporous materials for drug delivery. Microporous Mesoporous Mater..

[3] Slowing I.I., Vivero-Escoto J.L., Wu C.-W., Lin V.S.-Y. (2008). Mesoporous silica nanoparticles as controlled release drug delivery and gene transfection carriers. Adv. Drug Deliv. Rev..

[4] Pankhurst Q.A., Thanh N.T.K., Jones S.K., Dobson J. (2009). Progress in applications of magnetic nanoparticles in biomedicine. J. Phys. D.

[5] Tietze R., Lyer S., Dürr S., Alexiou C. (2012). Nanoparticles for cancer therapy using magnetic forces. Nanomedicine.

[6] Horcajada P., Rámila A., Férey G., Vallet-Regí M. (2006). Influence of superficial organic modification of MCM-41 matrices on drug delivery rate. Solid State Sci..

[7] Mahmoudi M., Sant S., Wang B., Laurent S., Sen T. (2011). Superparamagnetic iron oxide nanoparticles (SPIONs): Development, surface modification and applications in chemotherapy. Adv. Drug Deliv. Rev..

[8] Popova M., Trendafilova I., Szegedi Á., Momekova D., Mihály J., Momekov G., Kiss L.F., Lázár K., Koseva N. (2018). Novel SO_3_H functionalized magnetic nanoporous silica/polymer nanocomposite as a carrier in a dual-drug delivery system for anticancer therapy. Microporous Mesoporous Mater..

[9] Peralta M.E., Jadhav S.A., Magnacca G., Scalarone D., Mártire D.O., Parolo M.E., Carlos L. (2019). Synthesis and in vitro testing of thermoresponsive polymer-grafted core-shell magnetic mesoporous silica nanoparticles for efficient controlled and targeted drug delivery. J. Colloid Interface Sci..

[10] Teng Y., Du Y., Shi J., Pong P.W.T. (2020). Magnetic iron oxide nanoparticle-hollow mesoporous silica Spheres:Fabrication and potential application in drug delivery. Curr. Appl. Phys..

[11] Dorlo T.P.C., Balasegaram M., Beijnen J.H., de Vries P.J. (2012). Miltefosine: A review of its pharmacology and therapeutic efficacy in the treatment of leishmaniasis. J. Antimicrob. Chemother..

[12] Zhu Y.-B., Zhang Q., Zou J.-J., Yu C.-X., Xiao D.-W. (2008). Optimizing high-performance liquid chromatography method with fluorescence detection for quantification of tamoxifen and two metabolites in human plasma: Application to a clinical study. J. Pharm. Biomed. Anal..

[13] Chevalier M.T., Rescignano N., Martin-Saldaña S., González-Gómez Á., Kenny J.M., San Román J., Mijangos C., Álvarez V.A. (2017). Non-covalently coated biopolymeric nanoparticles for improved tamoxifen delivery. Eur. Polym. J..

[14] Day C.M., Hickey Y.S., Plush S., Gang S. (2020). Novel Tamoxifen Nanoformulations for Improving Breast Cancer Treatment: Old Wine in New Bottles. Molecules.

[15] Haidary S.M., Mohammed A.B., Córcoles E.P., Ali N.K., Ahmad M.R. (2016). Effect of coatings and surface modification on porous silicon nanoparticles for delivery of the anticancer drug tamoxifen. Microelectron. Eng..

[16] Pradhan L., Srivastava R., Bahadur D. (2014). pH- and thermosensitive thin lipid layer coated mesoporous magnetic nanoassemblies as a dual drug delivery system towards thermochemotherapy of cancer. Acta Biomater..

[17] Xuan Q.-J., Wang J.-X., Nanding A., Wang Z.-P., Liu H., Lian X., Zhang Q.-Y. (2014). Tumor-Associated Macrophages are Correlated with Tamoxifen Resistance in the Postmenopausal Breast Cancer Patients. Pathol. Oncol. Res..

[18] Batool A., Arshad R., Razzaq S., Nousheen K., Kiani M.H., Shahnaz G. (2020). Formulation and evaluation of hyaluronic acid-based mucoadhesive self nanoemulsifying drug delivery system (SNEDDS) of tamoxifen for targeting breast cancer. Int. J. Biol. Macromol..

[19] Kim W., Suh C.-Y., Cho S.-W., Roh K.-M., Kwong H., Song K., Shon I.-J. (2012). A new method for the identification and quantification of magnetite–maghemite mixture using conventional X-ray diffraction technique. Talanta.

[20] Mascolo M.C., Pei Y., Ring T.A. (2013). Room Temperature Co-Precipitation Synthesis of Magnetite Nanoparticles in a Large pH Window with Different Bases. Materials.

[21] Huh S., Wiench J.W., Yoo J.-C., Pruski M., Lin V.S.-Y. (2003). Organic Functionalization and Morphology Control of Me soporous Si licas via a Co-Condensat ion Synthe s is Method. Chem. Mater..

[22] Kresse G., Hafner J. (1994). Ab initio molecular-dynamics simulation of the liquid-metal--amorphous-semiconductor transition in germanium. Phys. Rev. B.

[23] Kresse G., Furthmüller J. (1996). Efficiency of ab-initio total energy calculations for metals and semiconductors using a plane-wave basis set. Comput. Mater. Sci..

[24] Perdew J.P., Burke K., Ernzerhof M. (1996). Generalized Gradient Approximation Made Simple. Phys. Rev. Lett..

[25] Grimme S. (2006). Semiempirical GGA-type density functional constructed with a long-range dispersion correction. J. Comput. Chem..

[26] Blöchl P.E. (1994). Projector augmented-wave method. Phys. Rev. B.

[27] Kresse G., Joubert D. (1999). From ultrasoft pseudopotentials to the projector augmented-wave method. Phys. Rev. B.

[28] Mosmann T. (1983). Rapid colorimetric assay for cellular growth and survival: Application to proliferation and cytotoxicity assays. J. Immunol. Methods.

[29] Konstantinov S.M., Elbl H., Berger M.R. (1999). BCR-ABL influences the antileukaemic efficacy of alkylphosphocholines. Br. J. Haematol..

[30] Flores-Holguin N. (2017). Theoretical Calculation of UV-Vis, IR Spectra and Reactivity Properties of Tamoxifen Drug: A Methodology Comparison. MOJ. Bioorg. Org. Chem..

[31] Socrates G. (2004). Infrared and Raman Characteristic Group Frequencies: Tables and Charts.

