# Supplementary material for: Tamoxifen Delivery System Based on PEGylated Magnetic MCM-41 Silica
Source: Molecules. 2020 Nov 4;25(21):5129. doi: 10.3390/molecules25215129 (PMC7663855; doi:10.3390/molecules25215129)
Supplement: Supplementary file 1 [file molecules-25-05129-s001.pdf]

Table S1. Relative and binding energies (in kJ/mol) and selected distances (in pm) for the modeled structures of tamoxifen complexes with silicate surfaces terminated by silanol groups or modified with -CH_2COOH groups.

Structure	ΔE	BE	H_{Ph}–O_{sub}[d]	O_{Ph}–H_{sub}[e]	$H_{methylene}$–O_{sub}[f]	N–H_{sub}	H_{amin}–O_{sub}[g]
T1	0						
T2	13						
T3	24						
T4	24						
T5	28						
T6	29						
T1_OH_1	52	-214	242;250;253;268		237;257;285	141	238;282
T1_OH_2	84	-182	244;256		258	157	260;284
T1_OH_3	131	-136	251[c];264; 274[c];289;297	229	253		263[c];273
T1_OH_4	148	-118	243;250;251	181			244
T1_OH_5	192	-75	251		258		262;287
T2_OH_1[b]	0	-279	232;233;242;252;260;263	189	260;262;263	161	237;255
T2_OH_2[b]	14	-265	215;254;264;272;289;293	269;291		156	
T3_OH_1[b]	131	-159	259;271	258			241
T3_OH_2[b]	151	-139	219;233	200			261;286
T1_COOH_1	0	-184	238;270[a];285;285;293		256;275[h];292[h]		243;270;271;273
T1_COOH_2	40	-144	232;270;278;291		301		259
T1_COOH_3	48	-136	235;265	273			249;268;277;295
T1_COOH_4	60	-123	240;250;271;273[a];286		257[a,h];259[a]		254;261
T1_COOH_5	63	-120	260;261;273;288;289;295		273		264;298
T1_COOH_6	124	-60	264;296[a]				255;257;265;275
T2_COOH_1[b]	36	-161	235;256;275;297[a];299;301				255[a];261;282[a],298
T2_COOH_2[b]	90	-106	246;247;248[a];249[a];262[a];286[a]	186	246		247[a];264;269
T3_COOH_1[b]	69	-138	260	296			266;292
T3_COOH_2[b]	78	-130	235;243[a];244[a];270[a];274[a];276[a]	298	260		253;283

[a] interaction with the O from the carbonyl part of the COOH groups
[b] BE is calculated with respect to the conformer denoted in the name of the structure – T2 and T3
[c] interaction of H atoms from the drug molecule with O centers from the silicate bound to two Si centers (Si-O-Si)

[d] distance between H atom from phenyl group and O center from COOH or SiOH from the support
[e] distance between phenolic O center from the drug molecule and a H atom from the CH_2COOH or SiOH part of the supports
[f] distance between H atom from methylene groups or methyl moiety of the ethyl group of the drug molecule and O center from carboxyl or silanol group
[g] distance between H atom from amino methyl or methylene groups of the drug molecule and O center from carboxyl or silanol group
[h] interaction includes H center from methylene group bound to the ether O center

Table S2. Selected calculated vibrational frequencies for three of the modeled structures of tamoxifen conformers and their complexes with silicate surfaces terminated by silanol groups or modified with $-CH_2COOH$ groups. The most stable complexes are marked with bold.

Structure	ν(ArC-H)	ν(C-H)	ν(C=C)[a]	ν(ArC=C)	δ(CH_2,CH_3)	ν(C-O)[c]	ν(C-N)	γ(ArC-H)
T1	3143-3091	3048-2816	1609;1585	1593-1474;1428-1324	1464-1351	1220;1008	1260;1174;1041;1034	981-686
T2	3182-3088	3048-2799	1610;1585	1594-1472;1427-1333	1461-1350	1236;1034	1270;1180;1048;1041	983-669
T3	3209-3095	3047-2806	1609;1585	1593-1472;1426-1328	1460-1351	1230;1008	1264;1170;1041;1036	981-665
T1_OH_1	3142-3088	3077-2929	1592;1579	1590-1478;1471-1322	1462-1354	1205;1030	1250;1182;1021;1002	975-682
T1_OH_2	3141-3080	3077-2898	1596;1586	1593-1474;1426-1326	1472-1353	1215;1035	1250;1179;1024;1017	992-684
T1_OH_3	3159-3080	3054-2738	1595;1583	1592-1473;1428-1329	1464-1352	1207;992	1268;1178;1045;1030	996-683
T1_OH_4	3145-3087	3044-2841	1597;1589	1593-1473;1430-1330	1462-1357	1198;992	1265;1174;1043;1034	980-685
T1_OH_5	3130-3084	3047-2785	1596;1583	1592-1473;1428-1325	1462-1357	1219;1008	1261;1171;1037;1027	980-682
**T2_OH_1**	**3149-3109**	**3109-2892**	**1608;1596**	**1596-1472;1425-1335**	**1475-1327**	**1243;1233;1041**	**1255;1183;1035;1019**	**989-685**
T2_OH_2	3144-3082	3069-2895	1615	1597-1472;1427-1332	1463-1354	1243;1229;1040	1260;1174;1040[b];1020	979-688
T3_OH_1	3157-3076	3042-2823	1611	1594-1475;1426-1331	1470-1346	1232;1002	1258;1180;1040;1032	982-689
T3_OH_2	3178-3090	3052-2817	1603	1593-1472;1426-1318	1465-1343	1211;984	1265;1178;1038;1038	992-698
**T1_COOH_1**	**3142-3087**	**3069-2846**	**1584**	**1599-1472;1426-1329**	**1465-1354**	**1227;1000**	**1262;1183;1037;1026**	**984-680**
T1_COOH_2	3154-3081	3055-2829	1600	1594-1473;1429-1331	1468-1354	1217;1006	1258;1173;1041;1033	981-684
T1_COOH_3	3157-3086	3048-2844	1598;1587	1593-1471;1429-1330	1461-1354	1222;1000	1262;1179;1038;1031	982-698
T1_COOH_4	3141-3087	3061-2822	1599;1597	1599-1473;1425-1326	1460-1352	1225;1004	1254;1180;1040;1030	983-684
T1_COOH_5	3144-3086	3069-2832	1595	1595-1473;1426-1320	1465-1359	1219;1005	1260;1173;1036;1032	989-685
T1_COOH_6	3149-3086	3045-2836	1598;1588	1594-1474;1427-1326	1465-1353	1215;1000	1242;1172;1039;1033	982-683
T2_COOH_1	3174-3092	3064-2806	1607	1596-1471;1428-1339	1461-1344	1250;1041	1265;1182;1044;1035	967-695
T2_COOH_2	3208-3091	3065-2789	1610;1586	1596-1471;1466-1331	1466-1349	1214;1016	1264;1180;1051;1036	984-694
T3_COOH_1	3169-3079	3053-2802	1614	1594-1470;1426-1327	1466-1344	1221;1012	1260;1177;1045;1030	981-697
T3_COOH_2	3147-3088	3059-2841	1603	1594-1471;1426-1327	1468-1355	1222;1001	1265;1166;1038;1033	989-686

[a] vibration of the alkene bond; the lower frequency is mixed with C=C stretching vibrations in aromatic rings
[b] mixed with C(CH_2)-O stretching vibration
[c] Higher frequencies correspond to C(Ph)-O stretchings while lower ones to the C(CH_2)-O stretchings

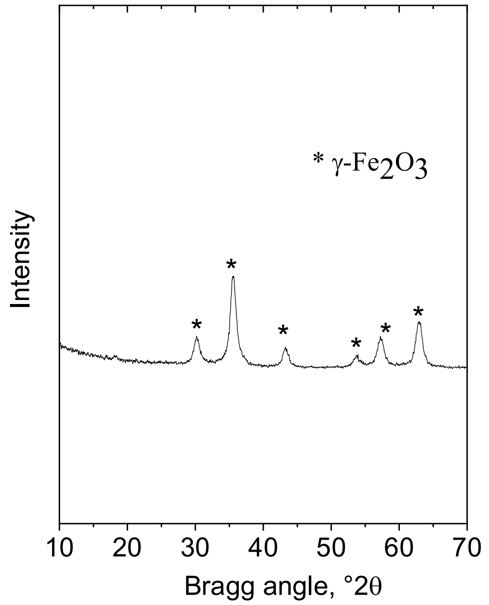

Figure S1. XRD of the iron oxide nanoparticles.

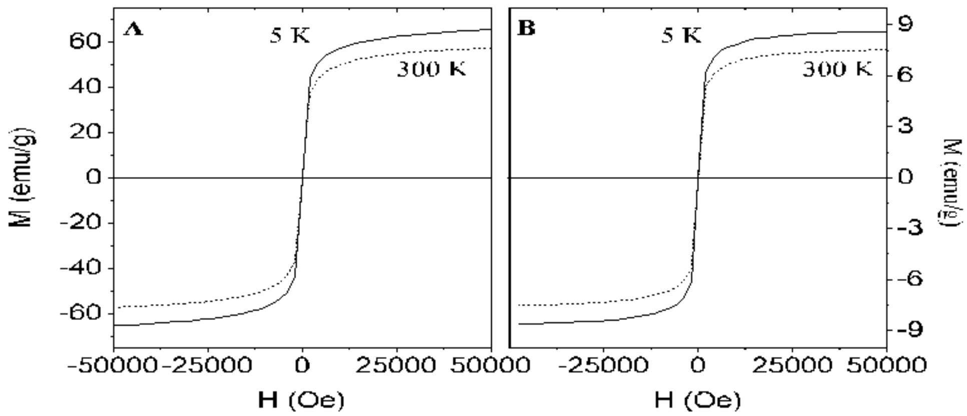

Figure S2. Magnetic properties of iron oxide nanoparticles and MM composite.

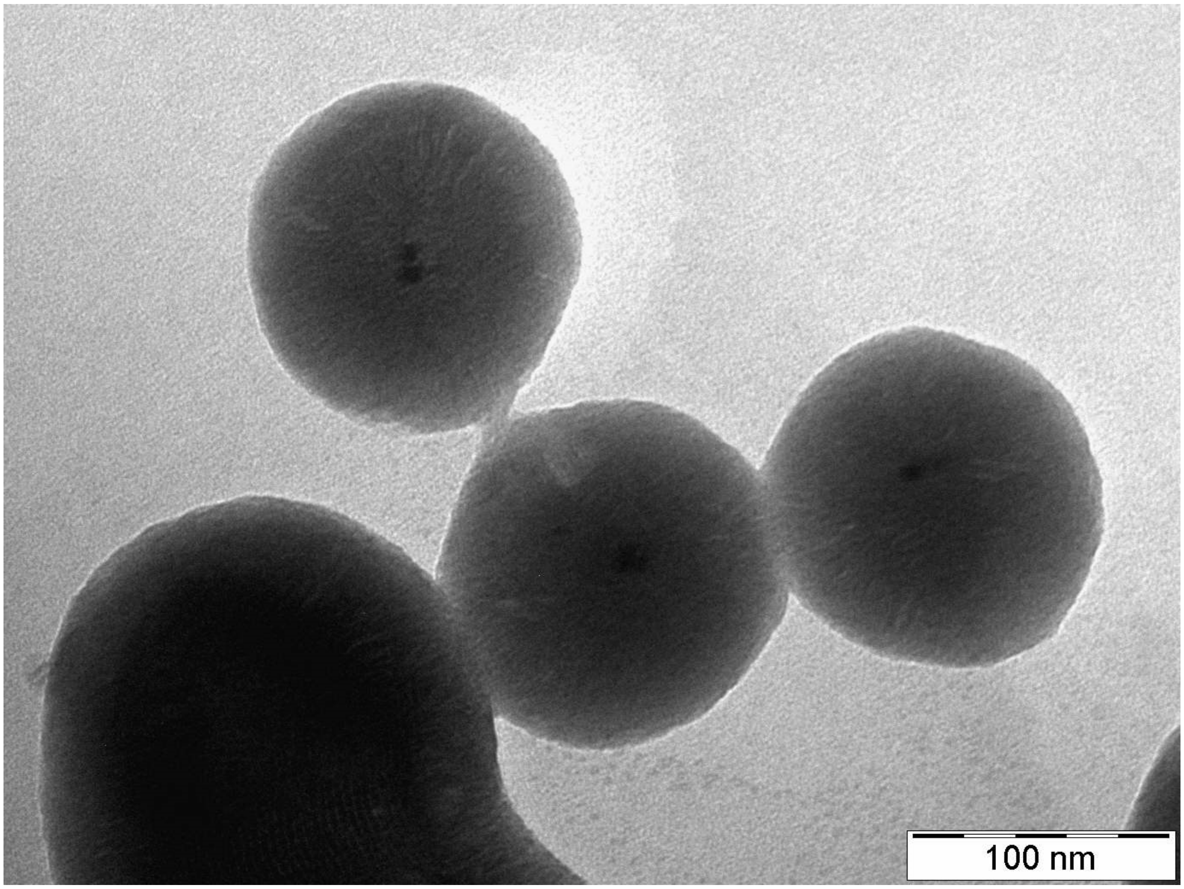

Figure S3. TEM images of MM composite.

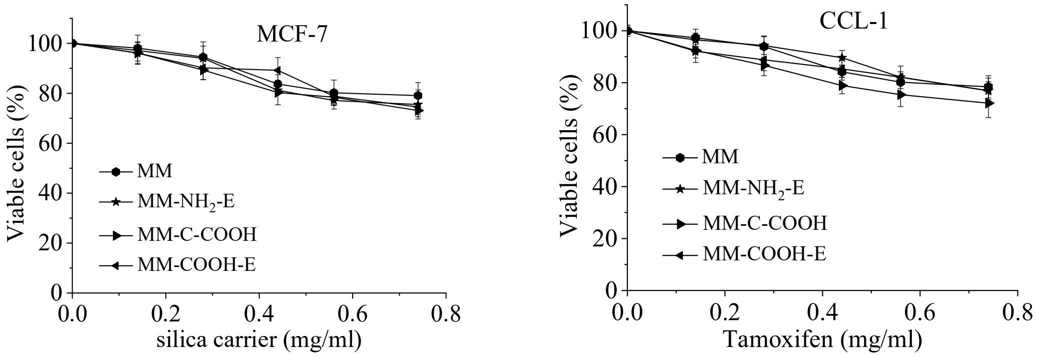

Figure S4. Cytotoxicity of the studied silica carriers against MCF-7 and CCL-1 cells after 72 h continuous exposure at 37°C. Each data point represents the arithmetic mean ± SD of 6 separate experiments.
